# Assessment of Treatment Strategies to Achieve Hepatitis C Elimination in Canada Using a Validated Model

**DOI:** 10.1001/jamanetworkopen.2020.4192

**Published:** 2020-05-06

**Authors:** Mawuena Binka, Naveed Z. Janjua, Jason Grebely, Chris Estes, Dena Schanzer, Jisoo A. Kwon, Naglaa H. Shoukry, Jeffrey C. Kwong, Homie Razavi, Jordan J. Feld, Mel Krajden

**Affiliations:** 1 British Columbia Centre for Disease Control, Vancouver, Canada; 2 Canadian Network on Hepatitis C, Montreal, Quebec, Canada; 3 University of British Columbia, Vancouver, Canada; 4 Kirby Institute, University of New South Wales Sydney, Sydney, Australia; 5 Center for Disease Analysis, Lafayette, Colorado; 6 Centre de Recherche du Centre Hospitalier de l’Universite de Montreal, Montreal, Quebec, Canada; 7 ICES, Toronto, Ontario, Canada; 8 Toronto Centre for Liver Disease, University Health Network, Toronto, Ontario, Canada

## Abstract

**Importance:**

Achievement of the World Health Organization (WHO) target of eliminating hepatitis C virus (HCV) by 2030 will require an increase in key services, including harm reduction, HCV screening, and HCV treatment initiatives in member countries. These data are not available for Canada but are important for informing a national HCV elimination strategy.

**Objective:**

To use a decision analytical model to explore the association of different treatment strategies with HCV epidemiology and HCV-associated mortality in Canada and to assess the levels of service increase needed to meet the WHO elimination targets by 2030.

**Design, Setting, and Participants:**

Study participants in this decision analytical model included individuals with hepatitis C virus infection in Canada. Five HCV treatment scenarios (optimistic, very aggressive, aggressive, gradual decrease, and rapid decrease) were applied using a previously validated Markov-type mathematical model. The optimistic and very aggressive treatment scenarios modeled a sustained annual treatment of 10 200 persons and 14 000 persons, respectively, from 2018 to 2030. The aggressive, gradual decrease, and rapid decrease scenarios assessed decreases in treatment uptake from 14 000 persons to 10 000 persons per year, 12 000 persons to 8500 persons per year, and 12 000 persons to 4500 persons per year, respectively, between 2018 and 2030.

**Main Outcomes and Measures:**

Hepatitis C virus prevalence and HCV-associated health outcomes were assessed for each of the 5 treatment scenarios with the goal of identifying strategies to achieve HCV elimination by 2030.

**Results:**

An estimated mean 180 142 persons (95% CI, 122 786-196 862 persons) in Canada had chronic HCV infection at the end of 2017. The optimistic and gradual decrease scenarios estimated a decrease in HCV prevalence from 180 142 persons to 37 246 persons and 37 721 persons, respectively, by 2030. Relative to 2015, this decrease in HCV prevalence was associated with 74%, 69%, and 69% reductions in the prevalence of decompensated cirrhosis, hepatocellular carcinoma, and liver-associated mortality, respectively, leading to HCV elimination by 2030. More aggressive treatment uptake (very aggressive scenario) could result in goal achievement up to 3 years earlier than 2030, although a rapid decrease in the initiation of treatment (rapid decrease scenario) would preclude Canada from reaching the HCV elimination goal by 2030.

**Conclusions and Relevance:**

The study findings suggest that Canada could meet the WHO goals for HCV elimination by 2030 by sustaining the current national HCV treatment rate during the next decade. This target will not be achieved if treatment uptake is allowed to decrease rapidly.

## Key Points

**Question:**

What strategies are needed to meet the goal of hepatitis C virus elimination in Canada by 2030?

**Findings:**

In this decision analytical model, the sustained annual treatment of 10 200 individuals with hepatitis C virus infection was associated with a 5-fold reduction in chronic hepatitis C virus infections and a substantial decrease in liver-associated morbidity and mortality, and the achievement of hepatitis C elimination in Canada by 2030. However, this model indicates that a rapid decrease in the initiation of hepatitis C virus treatment would preclude Canada from achieving this goal.

**Meaning:**

Results of this study suggest that hepatitis C virus elimination may be achievable in Canada by 2030 if current national hepatitis C virus treatment rates are sustained during the next decade.

## Teaser

This decision analytical model study explores the association of different treatment strategies with hepatitis C virus epidemiology and associated mortality among individuals with hepatitis C virus infection in Canada and assesses whether Canada can achieve hepatitis C virus elimination by 2030.

## Introduction

Chronic hepatitis C virus (HCV) infection is a substantial global health concern, with approximately 71 million people with chronic HCV infection worldwide.^1^ Hepatitis C virus infection is associated with many adverse health outcomes, including liver disease and death.^2^ Despite the availability of curative treatment since the early 2000s, complications associated with HCV infection continue to increase.^3,4,5^ The introduction of short-course highly effective direct-acting antiviral (DAA) treatments was a medical breakthrough that changed the management of HCV infection.^6,7,8^ Similar to interferon-based treatment, sustained virologic response (SVR) or HCV cure with newer DAA medications is associated with reductions in all-cause mortality and a lower risk of progression to advanced liver disease.^5,7,8,9,10^ Therefore, with adequate uptake of curative treatments, the global HCV disease burden could be substantially reduced.

The advent of DAA medications led the World Health Organization (WHO) to issue its first global health sector strategy on viral hepatitis, promoting HCV elimination through an 80% reduction in HCV infection incidence and a 65% reduction in HCV-associated deaths by 2030.^8^ To achieve these outcomes, improved access to testing and treatment as well as harm reduction, mental health support, and addiction support will be required. The service targets set by the WHO include the diagnosis of 90% of persons with chronic HCV infection and the treatment of 80% of those with HCV infection by 2030,^8^ raising the need for an estimation of the appropriate levels of service increase in various geographic regions.

The Canadian government has committed to reaching the HCV elimination goals^11^ as part of its pan-Canadian framework for action against sexually transmitted and blood-borne infections.^12^ However, until 2019, a detailed plan for HCV elimination efforts, including the levels of service increase needed to achieve these targets, had not been developed. The Canadian Network on Hepatitis C led the effort to develop a national blueprint for HCV elimination in Canada that will support the national framework for sexually transmitted and blood-borne infections.^13^ As part of these efforts, clinicians, researchers, public health experts, and policy makers conducted a modeling exercise to understand and estimate the services needed to achieve elimination targets.^14^ This article describes the association of different treatment strategies with HCV epidemiology and HCV-associated mortality in Canada to assess whether Canada can meet the WHO elimination targets by 2030.

## Methods

### HCV Health Policy Tool

The HCV Health Policy Tool, a mathematical model developed by the Center for Disease Analysis, was used to estimate the association between different treatment strategies and various HCV-associated health outcomes, including decompensated cirrhosis, hepatocellular carcinoma, and liver-associated death, for people with chronic HCV infection in Canada.^1^ The tool tracks and estimates disease progression among individuals with viremic HCV infection by age and sex in specific geographic regions.^15^ This Markov-type model was built using Microsoft Excel software (Microsoft Corp) and has been used by many countries to assess HCV prevalence and potential interventions that can be performed to achieve HCV elimination.^1,16,17,18,19,20,21^ The model has been described in detail elsewhere.^1,19^ The HCV disease burden in Canada was previously assessed with this model,^15^ which was updated with recent estimates for this analysis. Data used in the development of the HCV Health Policy Tool were publicly available and did not require ethics approval from an institutional review board in accordance with Article 2.2 of the Tri-Council Policy Statement: Ethical Conduct for Research Involving Humans (joint policy of Canada’s 3 federal research agencies: the Canadian Institutes of Health Research, the Natural Sciences and Engineering Research Council of Canada, and the Social Sciences and Humanities Research Council of Canada).^22^ Reporting was performed according to relevant noncost aspects of the Consolidated Health Economic Evaluation Reporting Standards (CHEERS) guideline for decision analytical model studies.^23^

### Inputs and Parameter Estimates

Canadian population data, organized by year, sex, and 5-year age groupings, were obtained from the United Nations population database (eTable 1 in the Supplement).^24^ Prevalence estimates for HCV infection were obtained from published Canadian data,^25,26^ and corresponding sex and 5-year age distributions were derived from Public Health Agency of Canada reports (eTable 2 in the Supplement).^27^ A viremic rate of 77% was applied to prevalence estimates. Viremia was defined as a positive test result for the presence of HCV ribonucleic acid.^28^

A national 1-day meeting was held in 2018 with the aim of developing expert consensus. During the meeting, experts from the Canadian Network on Hepatitis C gathered with experts in public health and national and provincial public health modeling; presentations regarding key parameters were given, which was followed by discussion of the various parameters to achieve consensus. Based on this expert consensus as well as national surveys, an estimated 30% of individuals with HCV infection were presumed to be undiagnosed in 2013.^15,29^ The number of new diagnoses from 1991 to 2016 was obtained from the Public Health Agency of Canada,^27^ with the number of viremic cases set at 77% of reported values.^28^ An average of 8378 persons with viremic HCV infection were newly diagnosed annually in Canada from 2014 to 2016,^27^ representing an annual increase of 4% within that period. In keeping with this trend, for all treatment scenarios, we estimated a 3% increase in new diagnoses from 2017 to 2018, a 2% increase in new diagnoses from 2019 to 2020, and a gradual decrease in new diagnoses to 50% of the 2020 diagnosis rate by 2025, as persons with HCV infection become progressively harder to reach (eTable 3 in the Supplement).

Annual age-specific incidence distributions were calculated to fit known prevalence distributions by age and sex.^26,27^ Based on the expert consensus of the Canadian Network on Hepatitis C collaborators, an estimated 2500 new HCV infections per year occurred in Canada from 2015 to 2017.^14^ New infections for subsequent years were set to achieve the required 30% reduction in HCV infection incidence by 2020 and the required 80% reduction for all treatment scenarios by 2025 (eTable 4 in the Supplement).

The disease progression framework of the model is illustrated in eFigure 1 in the Supplement, with transition probabilities detailed in Table 1. Consistent with previous models, a 23% (95% CI, 15%-45%) spontaneous clearance rate was assumed among persons with acute HCV infection.^30^ The annual age- and sex-specific liver disease progression rates that were applied in this model were published previously.^1,15^

**Table 1.** Model Inputs and Parameter Estimates

Parameter^a^	Value (range)	Source
Viremic HCV rate, %	77.0	Seeff,^28^ 2002
Disease progression rate, %		
Acute HCV to spontaneous clearance	23.0 (15.0-45.0)	Seeff et al,^30^ 2001
Acute HCV to F0	77.0	Kwon et al,^21^ 2019; Harris et al,^31^ 2014
F0 to F1	(4.4-21.8)	Kwon et al,^21^ 2019; Harris et al,^31^ 2014
F1 to F2	(3.4-14.3)	Kwon et al,^21^ 2019; Harris et al,^31^ 2014
F2 to F3	(4.5-22.4)	Kwon et al,^21^ 2019; Harris et al,^31^ 2014
F3 to F4	(4.7-20.0)	Kwon et al,^21^ 2019; Harris et al,^31^ 2014
F3 to HCC	0.2	Kwon et al,^21^ 2019; Bernfort et al,^32^ 2006
F4 to DC	3.0	Kwon et al,^21^ 2019; Bernfort et al,^32^ 2006
F4 to HCC	3.6	Kwon et al,^21^ 2019; Bernfort et al,^32^ 2006
DC to liver-associated death	20.0	Kwon et al,^21^ 2019; Bernfort et al,^32^ 2006; Ries et al,^33^ 2007
HCC to liver-associated death		
First year	70.7	Kwon et al,^21^ 2019; Bernfort et al,^32^ 2006; Ries et al,^33^ 2007
Subsequent years	16.2	Kwon et al,^21^ 2019; Bernfort et al,^32^ 2006; Ries et al,^33^ 2007
Reduction in progression rate from F3 or F4 to HCC among cured individuals	77.0	Kwon et al,^21^ 2019; Morgan et al,^34^ 2013
Reduction in progression rate from F4 to DC among cured individuals	76.0	Kwon et al,^21^ 2019; DiMarco et al,^35^ 2016; Nahon et al,^36^ 2017
Reduction in liver-associated mortality among cured individuals with DC and HCC	50.0	Kwon et al,^21^ 2019
Background death rate	1.8	Kwon et al,^21^ 2019; Human Mortality Database^37^
Standard mortality ratio		
Injection drug use	10.0 (9.5-29.9)	Frischer et al,^38^ 1997; Engstrom et al,^39^ 1991; Oppenheimer et al,^40^ 1994; Hickman et al,^41^ 2003; Bjornaas et al,^42^ 2008; Perucci et al,^43^ 1991
Transfusion	2.1 (1.3-17.6)	Kamper-Jorgensen et al,^44^ 2008

Abbreviations: DC, decompensated cirrhosis; F0, stage 0 fibrosis (no fibrosis); F1, stage 1 fibrosis (portal fibrosis without septa); F2, stage 2 fibrosis (portal fibrosis with few septa); F3, stage 3 fibrosis (portal fibrosis with numerous septa but without cirrhosis); F4, stage 4 fibrosis (cirrhosis); HCC, hepatocellular carcinoma; HCV, hepatitis C virus; SVR, sustained virologic response.

^a^ Additional input parameters (population size and age distribution, HCV prevalence, new viremic HCV diagnoses, new infections, number treated, and SVR rates) are presented in detail in eTables 1-3, 5, and 7 in the Supplement.

Cancer data, including hepatocellular carcinoma data, were obtained from Statistics Canada^45^ and were used to validate model results. The HCV-associated liver transplantation and background mortality data were obtained from the Canadian Organ Replacement Register^46^ and the Berkeley Human Mortality Database,^37^ respectively. Based on data from the British Columbia Hepatitis Testers Cohort,^47^ a standardized mortality ratio (SMR) of 10.0 (95% CI, 9.5-29.9) was estimated for persons aged 15 to 59 years who inject drugs, while the SMR for persons infected with HCV through blood transfusion was set at 2.1 (95% CI, 1.3-17.6).^15,19,38,39,40,41,42,44^

Annual age-associated and disease stage–associated treatment eligibility requirements were determined through consultation with experts in model design. Because more than 90% of people with chronic HCV infection in Canada had unrestricted access to publicly funded HCV treatment as of April 2018,^48^ fibrosis stage–independent access to HCV treatment was assumed for all individuals with HCV infection beginning in 2018. The HCV treatment uptake estimates before 2012 were calculated using pegylated interferon sales data from the IQVIA (formerly IMS Health and Quintiles) database.^15^ Treatment uptake estimates for interferon-based therapy from 2012 to 2017 were derived from IQVIA-reported prescription data, and uptake estimates for DAA treatment were based on the number of DAA pills dispensed and the recommended treatment regimens (Mawuena Binka, PhD, MPH, written communication, September 18, 2019). Genotype-specific HCV treatment durations and corresponding SVR rates were previously published and finalized with expert input (eTable 5 in the Supplement).^15,49,50,51,52,53,54,55^

The model was adjusted to account for the residual risk of decompensated cirrhosis, hepatocellular carcinoma, and liver-associated death after SVR,^5,9,56,57^ as delineated by Kwon et al.^21^ Fibrosis was categorized in 5 stages, with stage 0 indicating no fibrosis, stage 1 indicating portal fibrosis without septa, stage 2 indicating portal fibrosis with few septa, stage 3 indicating portal fibrosis with numerous septa but without cirrhosis, and stage 4 indicating cirrhosis. The model assumed a 77% reduction in progression rates from stage 3 or stage 4 fibrosis to hepatocellular carcinoma,^34^ a 76% reduction in progression from stage 4 fibrosis to decompensated cirrhosis,^35,36^ and a 50% reduction in progression to death^21^ for persons achieving SVR (Table 1).

### Treatment Scenarios

An estimated 5147 individuals began receiving HCV treatment in 2014, which is the year second-generation DAA medications became available in Canada (Mawuena Binka, PhD, MPH, written communication, September 18, 2019). This number doubled to approximately 12 718 persons by 2017. To evaluate appropriate treatment strategies to achieve HCV elimination in Canada by 2030 or earlier, HCV infection prevalence and HCV-associated liver disease were assessed in the context of 5 DAA treatment strategies—optimistic, very aggressive, aggressive, gradual decrease, and rapid decrease—that were developed in consultation with collaborators from the Canadian Network on Hepatitis C (Table 2). The optimistic and very aggressive treatment scenarios examined DAA treatment rates that were sustained at 10 200 persons per year or 14 000 persons per year, respectively, from 2018 to 2030. The aggressive scenario modeled high treatment uptake rates that decreased from 14 000 persons per year in 2018 to 10 000 person per year in 2030. The gradual decrease and rapid decrease scenarios examined decreases in treatment uptake rates from 12 000 persons per year in 2018 to 8500 persons per year or 4500 persons per year, respectively, in 2030.

**Table 2.** Treatment Scenarios, 2018 to 2030

Scenario	Persons treated per year
Very aggressive	14 000
Aggressive	Stepwise decrease from 14 000 to 10 000
Gradual decrease	Gradual decrease from 12 000 to 8500
Optimistic	10 200
Rapid decrease	Rapid decrease from 12 000 to 4500

Input parameters for treatment scenarios were entered into the model in 5 intervals, as summarized in eTable 6 and eTable 7 in the Supplement. With the exception of the number of treated individuals, all other input parameters were fixed for each treatment scenario. When multiple years were covered within an interval, the average value for the indicated interval was entered.

### Calibration and Sensitivity Analyses

The model has been previously validated for use with Canadian data.^15^ It was calibrated to an HCV infection prevalence of 233 432 persons (95% CI, 220 627-245 987 persons) in 2011.^25^ Beta-PERT distributions were generated for key assumptions within the model; assumptions included the number of new infections and new diagnoses in each interval, the transition probabilities across different fibrosis stages, and the SMR rates for persons who injected drugs or persons who acquired infections through blood transfusion. The number of new infections and diagnoses were set to vary between 20% above or below the estimate within each interval, while the upper and lower limits of the remaining variables were as indicated in Table 1.

The Oracle Crystal Ball add-in (Oracle Corp) for Excel software was used to perform 1000 Monte Carlo simulations and assess 95% CIs for the estimated number of liver-associated deaths and the number of persons with decompensated cirrhosis, hepatocellular carcinoma, and viremic HCV infection by 2030 for each treatment scenario. For the optimistic model, the leading factors in the uncertainty surrounding these estimates were also assessed.

## Results

An estimated mean 180 142 persons (95% CI, 122 786-196 862 persons) had chronic HCV infection at the end of 2017, which represented a decrease from the estimated mean of 204 707 persons with HCV infection in 2015. Within the 2017 group, approximately 34 755 persons (19%) had stage 0 fibrosis, 63 750 persons (35%) had stage 1 fibrosis, 29 345 persons (16%) had stage 2 fibrosis, 29 409 persons (16%) had stage 3 fibrosis, 18 955 persons (11%) had stage 4 fibrosis, 2241 persons (1%) had decompensated cirrhosis, 1176 persons (0.7%) had hepatocellular carcinoma, and 511 persons (0.3%) had undergone liver transplants (Table 3). An estimated 12 718 persons with HCV infection began receiving treatment in 2017, increasing the approximate number of persons treated since 2015 to 94 317 persons by year-end. Beginning with data from 2018, we modeled the 5 treatment strategies to achieve HCV elimination in Canada by 2030 (eTable 3 in the Supplement).

**Table 3.** HCV-Associated Health Outcomes in 2030 by Treatment Scenario^a^

Outcome	Mean (95% CI)					
	End of 2017	Optimistic	Aggressive	Gradual decrease	Rapid decrease	Very aggressive
HCV infection prevalence	180 142 (122 786-196 862)	37 246 (671-51 055)	26 291 (520-40 125)	37 721 (667-51 543)	59 618 (14 977-73 495)	1262 (436-10 987)
Fibrosis stage						
F0	34 755 (21 547-50 911)	5073 (522-8384)	4020 (464-6954)	5218 (521-8561)	7527 (2395-11 629)	805 (408-2447)
F1	63 750 (41 323-79 381)	8508 (49-14 422)	6044 (27-10 860)	8676 (44-14 587)	13 794 (3048-21 290)	158 (20-2765)
F2	29 345 (17 540-31 571)	5799 (8-8990)	4039 (1-6971)	5881 (4-9038)	9447 (1963-13 244)	76 (1-1808)
F3	29 409 (10 677-39 383)	8409 (9-10 916)	5780 (0-8225)	8480 (4-10 986)	13 640 (2839-16 587)	106 (0-2108)
F4	18 955 (5637-27 106)	7978 (11-10 045)	5390 (0-7379)	7983 (5-10 068)	12 856 (2421-15 374)	98 (0-1889)
Decompensated cirrhosis						
Total	2350 (623-3859)	838 (150-1149)	580 (132-836)	828 (150-1138)	1292 (274-1755)	252 (121-363)
Viremic HCV	2241 (554-3734)	630 (1-908)	363 (0-588)	621 (0-897)	1108 (130-1521)	6 (0-112)
Cured HCV	110 (71-145)	208 (110-294)	217 (113-307)	207 (109-292)	184 (99-258)	247 (120-336)
Hepatocellular carcinoma						
Total	1310 (530-2282)	640 (188-893)	527 (176-714)	638 (187-896)	841 (276-1257)	314 (160-426)
Viremic HCV	1176 (421-2104)	380 (1-618)	254 (0-421)	379 (1-616)	611 (116-988)	5 (0-84)
Cured HCV	135 (91-174)	260 (144-358)	273 (148-374)	259 (142-356)	231 (130-315)	309 (160-417)
Liver transplant	511 (288-563)	470 (1-514)	398 (1-514)	483 (1-525)	635 (215-660)	8 (0-129)
Liver-associated death						
Total	1215 (400-1788)	755 (249-939)	636 (227-803)	752 (249-936)	966 (334-1201)	434 (207-599)
Viremic HCV	1124 (338-1674)	389 (0-504)	253 (0-355)	387 (0-506)	640 (103-796)	5 (0-79)
Cured HCV	91 (61-114)	466 (189-515)	384 (195-541)	365 (188-512)	327 (173-453)	430 (206-595)
Year of outcome achievement						
90% diagnosed with HCV^b^		2022	2022	2022	2022	2022
80% treated for HCV		2030	2028	2030	2034	2027
80% decrease in HCV incidence^b^		2025	2025	2025	2025	2025
65% decrease in liver-associated death						
Viremic HCV only		2030	2028	2030	2034	2026
Viremic and cured HCV		2034	2033	2034	2040	2030
Year of all target outcome achievement						
Viremic HCV only		2030	2028	2030	2034	2027
Viremic and cured HCV		2034	2033	2034	2040	2030

Abbreviations: F0, stage 0 fibrosis (no fibrosis); F1, stage 1 fibrosis (portal fibrosis without septa); F2, stage 2 fibrosis (portal fibrosis with few septa); F3, stage 3 fibrosis (portal fibrosis with numerous septa but without cirrhosis); F4, stage 4 fibrosis (cirrhosis); HCV, hepatitis C virus.

^a^ Best estimate.

^b^ Fixed within model.

**Figure 1.**
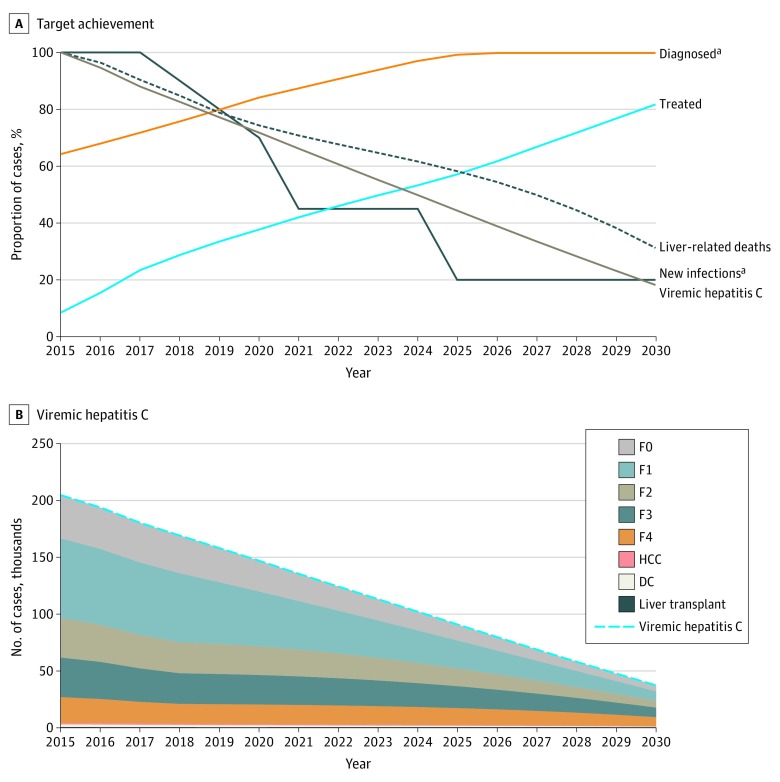
Optimistic Scenario for Achievement of WHO Targets by 2030 A, Target achievement. The numbers of individuals with viremic hepatitis C, new hepatitis C viral infections, and liver-associated deaths are relative to 2015. B, Viremic hepatitis C. DC indicates decompensated cirrhosis; F0, no fibrosis; F1, portal fibrosis without septa; F2, portal fibrosis with few septa; F3, portal fibrosis with numerous septa but without cirrhosis; F4, cirrhosis; HCC, hepatocellular carcinoma; and WHO, World Health Organization. ^a^Fixed for all treatment scenarios.

**Figure 2.**
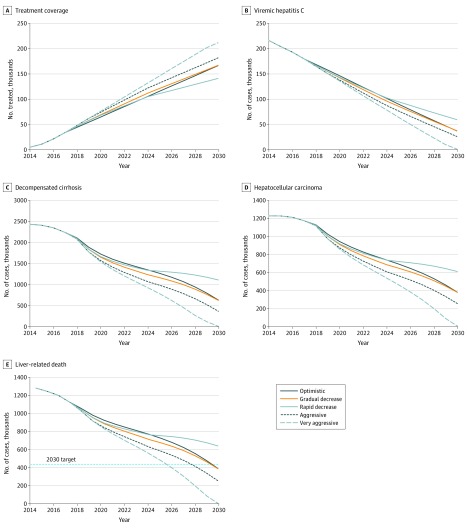
Hepatitis C Virus–Associated Outcomes by Treatment Scenario A, Treatment coverage. B, Viremic hepatitis C. C, Decompensated cirrhosis. D, Hepatocellular carcinoma. E, Liver-associated death.

The model estimated that the treatment of 10 200 persons with HCV infection annually between 2018 and 2030 (optimistic scenario) would result in an 82% reduction in the prevalence of chronic viremic HCV infection, from 204 707 persons in 2015 to 37 246 persons by 2030 (Figure 1 and Figure 2) (eFigure 2A in the Supplement). An estimated 166 952 persons (82%) with a diagnosis of HCV infection would receive treatment between 2015 and 2030, with substantial decreases in HCV infection prevalence if all individuals with HCV infection, regardless of disease stage, received treatment. In this scenario, liver-associated mortality would decrease 69%, from 1244 persons in 2015 to 389 persons in 2030, with corresponding decreases of 74% (2412 persons to 630 persons) in the prevalence of decompensated cirrhosis and 69% (1229 persons to 380 persons) in the prevalence of hepatocellular carcinoma (Figure 2) (eFigure 2 in the Supplement). Similar results were estimated with the gradual decrease scenario, in which the prevalence of chronic viremic HCV infection decreased from 204 707 persons in 2015 to 37 721 persons by 2030 (Figure 2 and Table 3). With the adoption of either of these strategies, given the fixed assumption of an 80% reduction in new HCV infections by 2025 and the diagnosis of 90% of persons infected with HCV by 2022 for all treatment scenarios, HCV elimination in Canada was achievable by the end of 2030 (Table 3).

Hepatitis C virus elimination earlier than 2030 was estimated with more aggressive approaches to increasing HCV treatment uptake in Canada. Target achievement by 2028 was estimated with the aggressive scenario, in which the stepwise decrease in treatment from 14 000 persons to 12 000 persons per year, followed by 10 000 persons per year, could lead to an estimated 79%, 85%, and 80% reduction in the prevalence of hepatocellular carcinoma, decompensated cirrhosis, and liver-associated death, respectively, by 2030 (Figure 2 and Table 3). An increase in the treatment rate to a sustained rate of 14 000 individuals per year from 2018 to 2030 (very aggressive scenario) would produce greater reductions in HCV-associated morbidity and liver-associated mortality and promote HCV elimination by 2027. However, the rapid decrease scenario, in which a rapid decrease in national treatment rates from 12 000 individuals per year in 2018 to 4500 individuals per year in 2030 occurred, would delay HCV elimination in Canada by approximately 4 years (Table 3).

When liver-associated deaths after SVR were also considered (eFigure 3 in the Supplement), all of the proposed treatment strategies, with the exception of the very aggressive scenario, did not achieve the required 65% reduction in liver-associated mortality by 2030 (eFigure 3C in the Supplement). The achievement of the HCV elimination goal was delayed by up to a decade, with the targeted reduction in liver-associated deaths being attained in 2033, 2034, 2034, and 2040 for the aggressive, optimistic, gradual decrease, and rapid decrease treatment scenarios, respectively (Table 3). With the very aggressive treatment strategy (14 000 persons per year), liver-associated deaths among persons with viremic and cured HCV infection would decrease to 33% in 2030, similar to that achieved only among persons with viremic HCV in the optimistic scenario (eFigure 3C in the Supplement).

In the optimistic scenario, more than 90% of the uncertainty surrounding the estimated prevalence of HCV infection, decompensated cirrhosis, hepatocellular carcinoma, and liver-associated death for 2030 was associated with assumptions pertaining to spontaneous clearance, SMRs for persons who inject drugs and persons who acquired HCV through blood transfusion, and HCV infection incidence in 2011 (data not shown). The transition probabilities for hepatocellular carcinoma to liver-associated death and for mild fibrosis to moderate fibrosis were additional factors in the uncertainty of estimates of number of individuals with HCV infection and hepatocellular carcinoma, respectively.

## Discussion

In this study, a mathematical model was used to assess the association between different treatment strategies and HCV-associated morbidity and mortality in Canada and to assess whether Canada could achieve the WHO goals of HCV elimination by 2030. Assuming unrestricted access to publicly funded HCV treatment for all persons with HCV infection who were 15 years or older in 2018^48^ and the achievement of the WHO targets for HCV diagnosis and infection incidence before 2030, our analyses indicated that HCV elimination could be attained in Canada by 2030 with the sustained annual treatment of 10 200 persons with HCV infection from 2018 to 2030. This goal could also be achieved with a gradual decrease in annual treatment initiation from 12 000 individuals per year to 8500 individuals per year within this period. The estimated benefits of HCV elimination in Canada by 2030 included an 82% reduction in total individuals with viremic HCV infection, a 74% decrease in individuals with decompensated cirrhosis, a 69% reduction in individuals with hepatocellular carcinoma, and a 69% decrease in liver-associated mortality compared with 2015. More aggressive treatment uptake strategies would hasten goal achievement by 2 to 3 years, resulting in substantial improvements in HCV-associated morbidity and mortality across the country. The model estimated that Canada would not achieve HCV elimination by the 2030 deadline if treatment initiation decreases rapidly from 2018 to 2030, delaying HCV elimination by a minimum of 4 years. In addition, HCV elimination goals could only be reached with the annual treatment of at least 14 000 individuals with HCV infection when liver-associated deaths after HCV cure were also considered.

Maintaining high treatment uptake rates over a 13-year period to achieve HCV elimination in Canada may prove challenging. An estimated 34 353 persons began receiving HCV treatment in Canada between 2015 and 2017, with an average of 11 451 persons receiving treatment each year (Mawuena Binka, PhD, MPH, written communication, September 18, 2019). These high levels of treatment uptake in recent years may be partly owing to treatment initiation among patients with HCV infection who deferred treatment until well-tolerated DAA therapies became available.^58,59^ Treatment rates may increase further with the removal of restrictions in drug coverage across Canada in 2018,^48,60,61,62^ boding well for the attainment of proposed treatment targets in the short term. However, the removal of treatment restrictions in Australia resulted in a 6-fold increase in HCV treatment initiation between 2015 and 2016 followed by a substantial decrease in subsequent years.^60,61^ Thus, HCV treatment rates in Canada could decrease below the proposed annual targets after the initial treatment rate increase if measures are not instituted to support the continued identification of treatment-eligible candidates, both among persons with chronic HCV infection who are not yet diagnosed and persons who are not currently receiving treatment for HCV infection, between now and 2030.^61^ A decrease in treatment initiation to pre-DAA treatment levels would not only prevent HCV elimination by 2030 but could also result in a resurgence of HCV-associated morbidity and mortality given the persistent risk of viral transmission by individuals with viremic HCV infection. Therefore, it is important that public health policies and programs be implemented to support the testing and diagnosis of an adequate number of individuals with HCV infection to meet and exceed annual treatment targets and to keep individuals with HCV infection engaged in care and committed to disease prevention beyond SVR.^19,20,61,63,64,65,66^ These programs may include initiatives to raise awareness of (1) the need for HCV screening and diagnosis, (2) the adverse health outcomes associated with untreated HCV infection, and (3) the availability of publicly funded HCV treatment among key at-risk groups.^13^ Also necessary would be the deployment of a variety of care models and technologies, such as point-of-care approaches and dried blood–spot testing, the simplification of testing and treatment processes, the expansion of settings for HCV management and types of health care professionals who are trained in HCV management, the improvement of patient connections to care through the use of more accessible resources, such as peer navigators, and the integration of addiction and HCV care.

Studies from the United States, Canada, and Europe have shown lower risk reduction for hepatocellular carcinoma and liver-associated mortality after DAA treatment of individuals at more advanced stages of disease.^5,9,56,57^ Thus, because a substantial proportion of treatment-eligible individuals with chronic HCV infection in Canada began receiving DAA treatment at advanced disease stages before 2018, hepatocellular carcinoma diagnoses may continue to increase among these individuals. In this study, the residual risk of disease progression after SVR among those diagnosed, treated, and cured at advanced stages of liver disease was estimated to preclude Canada from achieving the 2030 liver-associated mortality target, similar to findings in Australia.^21^ To achieve mortality targets within this context, Canada should treat 14 000 people each year from 2018 to 2030, the achievement of which will require substantial efforts. This finding highlights the importance of early HCV diagnosis for improved health outcomes with treatment and reinforces the need for engagement with care and continued surveillance for the development of hepatocellular carcinoma after SVR, especially among those diagnosed late in the course of their HCV infections.^67^ Lack of adoption of robust screening strategies to support early diagnoses and loss to follow-up after SVR could erode the benefits of treatment increases within subsets of the population and prevent Canada from eliminating HCV as a public health concern.

### Limitations

This study had several limitations. The assumption that the WHO incidence targets for HCV will be met by 2025 was a limitation that was necessary to facilitate the study of treatment strategies for HCV elimination in a nondynamic model. Because HCV infection incidence was a static input, the relationship between the various treatment strategies and HCV incidence was not assessed. Future iterations of this study could be enhanced by factoring in incidence rates of primary HCV infection and reinfection within hard-to-reach at-risk populations, such as persons who inject drugs. The consequences of opioid misuse for HCV infection incidence within these populations should also be considered, as the persistence or expansion of opioid misuse may undermine efforts to achieve the HCV elimination targets.^68^

The model was also limited by the accuracy of the various estimated input parameters built into its framework, including assumptions about disease progression and mortality. Furthermore, the model did not account for differing SVR rates among treatment-naive vs treatment-experienced individuals, although this factor was expected to have limited consequences for the findings given the high SVR rates after retreatment.^69,70^ The persistent risk of reinfection after SVR was also not addressed in this model and may have resulted in some overestimation of the association between treatment strategies and HCV infection prevalence.^71^ In addition, the model assumed uniform treatment initiation among all individuals with HCV infection and did not account for various barriers to treatment uptake among different risk groups, including persons who inject drugs, many of whom are not reached by health care services; however, this factor may have been partially accounted for by including treatment coverage by fibrosis level in the model.^72,73,74^

The model also did not consider the potential consequences of immigration on HCV infection prevalence estimates in the future, which are relevant given the Canadian government’s plans to admit approximately 1 million immigrants from 2019 to 2021 and the fact that many of these immigrants originate from regions with relatively higher HCV infection prevalence.^6,75^ The costs associated with the implementation of these treatment strategies were also not addressed in this study. The increase of HCV treatment has been reported to be cost-effective in many countries;^18,64,76^ therefore, understanding these costs within the Canadian context would be useful for policy-making. These financial issues were beyond the scope and capability of this project and should be addressed in future studies.

## Conclusions

In this analysis, we identified various treatment strategies that would allow Canada to reach the WHO targets for HCV elimination by 2030 or earlier. Canada can achieve this goal by either sustaining high treatment rates during the next decade or by taking the potentially more feasible approach of increasing treatment uptake in the early years to allow for a gradual decrease in uptake over time. Whichever approach is taken, the treatment strategies described in this study are reliant on the adoption of public health policies to support the screening and diagnosis of almost every individual with chronic HCV infection in Canada over a 13-year period. Studies in Australia and England have highlighted increased awareness of HCV among at-risk groups as a necessary first step for the successful implementation of any such public health strategy.^65,77^ Canadian provinces would need to increase capacity for testing and treatment to match the expected increase in demand for HCV-associated care that would be generated by successful educational campaigns.^77,78^ Thus, HCV elimination in Canada would require sustained efforts by various stakeholders to build the infrastructure necessary to support the diagnosis, connection to care, and annual treatment of more than 10 000 people with chronic HCV infection across the country until 2030. Reaching this goal would not only place Canada within the ranks of successful countries worldwide but would also serve as a demonstration of Canada’s strong commitment to the overall health and wellness of Canadians with chronic HCV infection.

## Notes

Supplement. **eTable 1.** Quinquennial Canadian Population by Sex and 5-Year Age Groups, 1950 to 2015 **eTable 2.** Reported Hepatitis C (HCV) Cases in Canada by Sex and 5-Year Age Groups, 2000 to 2015 **eTable 3.** Fixed Input Parameters: New HCV Diagnoses **eTable 4.** Fixed Input Parameters: Treatment Eligibility Requirements and New Infections **eTable 5.** Sustained Virologic Response Rates Applied in the Model **eTable 6.** Treatment Scenario Assumptions Entered Into the Model **eTable 7.** Treatment Scenarios: Annual Number of Treated Individuals **eFigure 1.** Flow Diagram of Disease Progression Within the Model **eFigure 2.** Projected Trends in HCV-Associated Outcomes With Each Treatment Scenario **eFigure 3.** Advanced Liver Disease Diagnoses Among Patients Achieving Sustained Virologic Response After HCV Treatment **eReferences.**
