# Supplementary material for: Assessment of Treatment Strategies to Achieve Hepatitis C Elimination in Canada Using a Validated Model
Source: JAMA Netw Open. 2020 May 6;3(5):e204192. doi: 10.1001/jamanetworkopen.2020.4192 (PMC7203608; doi:10.1001/jamanetworkopen.2020.4192)
Supplement: Supplement. — eTable 1. Quinquennial Canadian Population by Sex and 5-Year Age Groups, 1950 to 2015 eTable 2. Reported Hepatitis C (HCV) Cases in Canada by Sex and 5-Year Age Groups, 2000 to 2015 eTable 3. Fixed Input Parameters: New HCV Diagnoses eTable 4. Fixed Input Parameters: Treatment Eligibility Requirements and New Infections eTable 5. Sustained Virologic Response Rates Applied in the Model eTable 6. Treatment Scenario Assumptions Entered Into the Model eTable 7. Treatment Scenarios: Annual Number of Treated Individuals eFigure 1. Flow Diagram of Disease Progression Within the Model eFigure 2. Projected Trends in HCV-Associated Outcomes With Each Treatment Scenario eFigure 3. Advanced Liver Disease Diagnoses Among Patients Achieving Sustained Virologic Response After HCV Treatment eReferences. [file jamanetwopen-3-e204192-s001.pdf]

Supplementary Online Content

Binka M, Janjua NZ, Grebely J, et al. Assessment of treatment strategies to achieve hepatitis C elimination in Canada using a validated model. *JAMA Netw Open*. 2020;3(5):e204192. doi:10.1001/jamanetworkopen.2020

eTable 1. Quinquennial Canadian Population by Sex and 5-Year Age Groups, 1950 to 2015

eTable 2. Reported Hepatitis C (HCV) Cases in Canada by Sex and 5-Year Age Groups, 2000 to 2015

eTable 3. Fixed Input Parameters: New HCV Diagnoses

eTable 4. Fixed Input Parameters: Treatment Eligibility Requirements and New Infections

eTable 5. Sustained Virologic Response Rates Applied in the Model

eTable 6. Treatment Scenario Assumptions Entered Into the Model

eTable 7. Treatment Scenarios: Annual Number of Treated Individuals

eFigure 1. Flow Diagram of Disease Progression Within the Model

eFigure 2. Projected Trends in HCV-Associated Outcomes With Each Treatment Scenario

eFigure 3. Advanced Liver Disease Diagnoses Among Patients Achieving Sustained Virologic Response After HCV Treatment

eReferences

This supplementary material has been provided by the authors to give readers additional information about their work.

© 2020 Binka M et al. *JAMA Network Open.*

eTable 1. Quinquennial Canadian Population by Sex and 5-Year Age Groups, 1950-2015[1]

This table presents the estimated Canadian population size from 1950-2015, organized by year, sex and age groupings, as obtained from the United Nations population database.[1]

Sex	Age Group	1950	1955	1960	1965	1970	1975	1980	1985	1990	1995	2000	2005	2010	2015
Male[a]	0-4	835	992	1139	1154	963	909	920	948	988	1018	918	882	965	996
	5-9	671	886	1040	1155	1172	995	931	933	987	1021	1044	956	930	997
	10-14	571	701	911	1045	1171	1200	1014	943	964	1025	1055	1086	993	952
	15-19	544	576	702	902	1057	1198	1231	1042	995	1020	1078	1120	1141	1089
	20-24	552	563	587	694	935	1099	1225	1272	1083	1024	1061	1134	1217	1273
	25-29	550	600	619	606	773	1018	1104	1232	1301	1107	1053	1096	1215	1231
	30-34	513	586	643	630	656	821	1026	1112	1275	1322	1143	1101	1152	1261
	35-39	488	549	620	646	651	674	817	1022	1140	1289	1351	1190	1154	1188
	40-44	432	510	554	612	646	659	678	810	1039	1148	1303	1386	1220	1172
	45-49	378	443	507	536	606	636	645	660	808	1035	1149	1312	1406	1216
	50-54	338	375	432	489	517	595	619	629	656	792	1024	1147	1308	1401
	55-59	296	317	355	403	466	487	566	599	618	643	775	997	1129	1313
	60-64	263	265	286	322	373	435	453	530	572	595	616	755	967	1101
	65-69	221	236	238	251	287	332	386	407	490	526	552	578	715	944
	70-74	156	183	195	197	203	238	274	322	351	421	461	491	523	654
	75-79	92	111	131	139	141	148	177	206	250	272	336	380	417	453
	80+	65	83	103	124	139	145	155	181	-	-	-	-	-	-
	80-84	-	-	-	-	-	-	-	-	134	164	187	246	297	321
	85-89	-	-	-	-	-	-	-	-	58	70	88	106	150	189
	90-94	-	-	-	-	-	-	-	-	18	22	26	35	45	67
	95-99	-	-	-	-	-	-	-	-	4	4	5	6	9	12
	100+	-	-	-	-	-	-	-	-	1	1	1	1	1	1
Female[a]	0-4	801	951	1088	1096	920	862	874	900	942	968	876	835	917	948
	5-9	645	850	996	1106	1118	948	883	886	936	972	994	910	876	951
	10-14	554	673	872	1001	1121	1146	964	899	915	973	1003	1037	944	899
	15-19	534	564	678	875	1025	1154	1181	987	943	964	1019	1058	1088	1028

© 2020 Binka M et al. *JAMA Network Open.*

Sex	Age Group	1950	1955	1960	1965	1970	1975	1980	1985	1990	1995	2000	2005	2010	2015
Female[a]	20-24	558	559	591	699	924	1084	1202	1221	1046	989	1012	1091	1152	1221
	25-29	564	593	597	608	755	983	1098	1213	1268	1085	1025	1068	1184	1220
	30-34	521	599	630	611	637	788	999	1113	1262	1297	1124	1083	1151	1266
	35-39	478	549	628	637	622	648	786	996	1143	1285	1329	1167	1142	1200
	40-44	411	490	550	622	630	630	653	787	1021	1158	1303	1364	1202	1174
	45-49	353	411	487	537	617	627	625	647	793	1029	1166	1312	1388	1204
	50-54	318	347	407	478	527	619	622	623	649	794	1025	1167	1313	1390
	55-59	276	304	337	392	472	513	609	620	621	654	785	1018	1161	1313
	60-64	239	257	285	324	385	462	504	598	611	615	642	780	1005	1140
	65-69	197	223	243	271	315	371	445	487	582	589	593	623	758	979
	70-74	147	179	203	222	247	287	343	410	453	537	547	557	586	720
	75-79	92	111	136	159	184	208	246	296	359	392	471	488	503	536
	80+	80	102	127	160	198	236	284	344	-	-	-	-	-	-
	80-84	-	-	-	-	-	-	-	-	228	279	314	388	416	424
	85-89	-	-	-	-	-	-	-	-	123	151	186	215	276	304
	90-94	-	-	-	-	-	-	-	-	50	61	74	94	115	152
	95-99	-	-	-	-	-	-	-	-	13	16	19	24	32	41
	100+	-	-	-	-	-	-	-	-	2	2	3	3	4	6

[a]Population estimates in thousands

© 2020 Binka M et al. *JAMA Network Open.*

eTable 2. Reported Hepatitis C (HCV) Cases in Canada by Sex and 5-Year Age Groups, 2000-2015[2]

This table presents reported HCV cases from 2000-2015, organized by year, sex and age groupings, as obtained from the Public Health Agency of Canada.[2] HCV cases from 2000-2015 are provided, data from 1991-2015 are available.

	Age Group	2000	2001	2002	2003	2004	2005	2006	2007	2008	2009	2010	2011	2012	2013	2014	2015
Male[a]	< 1	42	25	31	22	24	19	13	16	15	22	12	11	8	6	6	11
	1-4	11	4	8	6	9	2	1	4	4	0	2	2	4	8	6	4
	5-9	7	10	8	6	6	4	2	4	4	5	2	5	3	2	0	4
	10-14	21	27	10	5	10	7	7	3	8	5	6	3	2	3	1	7
	15-19	108	110	82	69	56	76	66	77	66	58	67	71	68	72	66	68
	20-24	439	426	429	395	418	378	331	335	356	323	315	286	378	487	442	500
	25-29	808	731	635	611	656	614	558	566	583	587	499	516	504	562	624	712
	30-39	3419	3163	2754	2333	2154	1912	1646	1534	1400	1395	1255	1186	1216	1220	1150	1322
	40-59	5597	5762	5796	5512	5409	4887	4574	4362	4426	4116	3887	3486	3292	3146	2833	2912
	60+	678	703	652	581	581	563	555	616	644	607	708	807	883	1039	1095	1228
	Unspecified	64	29	31	32	10	15	10	6	7	11	6	3	2	4	368	1
Female[a]	< 1	34	17	20	27	18	18	21	17	15	16	18	8	11	7	13	11
	1-4	10	5	8	10	4	7	9	8	7	4	4	8	8	5	4	5
	5-9	9	3	5	4	7	2	1	4	4	1	4	2	5	2	3	9
	10-14	22	16	19	11	6	12	6	5	9	7	5	8	3	4	5	3
	15-19	188	163	166	146	126	141	130	128	142	143	100	91	112	128	119	140
	20-24	384	349	328	366	403	399	333	348	374	337	336	319	396	400	383	485
	25-29	487	475	429	424	454	379	442	439	430	418	391	387	453	495	462	539
	30-39	1740	1559	1421	1279	1213	1023	878	891	906	791	733	730	762	738	693	859
	40-59	2335	2370	2448	2293	2179	1977	1879	2022	1878	1773	1670	1625	1518	1432	1321	1361
	60+	695	697	597	575	554	503	478	476	506	466	441	487	501	563	567	641
	Unspecified	26	14	15	20	9	9	6	3	4	4	6	1	5	5	252	1

[a]2000-2015 provided, 1991-2015 available.

© 2020 Binka M et al. *JAMA Network Open.*

eTable 3. Fixed Input Parameters: New HCV diagnoses

This table presents the number of new HCV diagnoses as added to the model. For all treatment scenarios, a 3% increase in new diagnoses was predicted from 2017-2018, a 2% increase from 2019-2020, followed by a gradual decline to 50% of the 2020 diagnosis rate in 2025, as affected persons become progressively harder to reach. Projected, input and model output counts are provided, where the average projected values for 2021-2024 were input for each of those years.

Year[b]	New HCV diagnoses[a]						
	Projected	Input	Model Output				
			Optimistic	Aggressive	Gradual decrease	Rapid decrease	Very aggressive
**2015**	8,371	8,371	8,371	8,371	8,371	8,371	8,371
**2016**	8,674	8,674	8,674	8,674	8,674	8,674	8,674
**2017**	8,934	8,934	8,934	8,934	8,934	8,934	8,934
**2018**	9,202	9,202	9,202	9,202	9,202	9,202	9,202
**2019**	9,386	9,386	9,386	9,386	9,386	9,386	9,386
**2020**	9,574	9,574	9,574	9,574	9,574	9,574	9,574
**2021**	8,617	7,181[c]	7,181	7,181	7,181	7,181	7,181
**2022**	7,659	7,181[c]	7,181	7,181	7,181	7,181	7,181
**2023**	6,702	7,181[c]	7,181	7,181	7,181	7,181	7,181
**2024**	5,744	7,181[c]	7,181	7,181	7,181	7,181	7,181
**2025**	4,787	4,787	4,787	4,787	4,787	4,787	4,787
**2026**	4,787	4,787	1,609	1,693	1,644	1,619	1,733
**2027**	4,787	4,787	378	378	378	377	381
**2028**	4,787	4,787	384	384	384	383	384
**2029**	4,787	4,787	383	384	383	383	384
**2030**	4,787	4,787	384	384	384	383	384

[a] Viremic cases only; [b] 2015-2016 based on Public Health Agency of Canada data, 2017-2030 based on trend; [c]Average of 2021-2024.

© 2020 Binka M et al. *JAMA Network Open.*

eTable 4. Fixed Input Parameters: Treatment Eligibility Requirements and New Infections

This table presents the eligibility requirements entered into the model for indicated years, including age and fibrosis stage. Average SVR rates input for each year are also provided. Average projected number of new HCV infections for 2021-2024 were input for each of those years.

Year	Eligible Age	Average SVR rate	Medically Eligible	New infections	
				Projected	Input
**2015**	15-74	90%	≥F2	2,500	2,500
**2016**	15-85+	90%	≥F2	2,500	2,500
**2017**	15-85+	95%	≥F2	2,500	2,500
**2018**	15-85+	95%	≥F0	2,250	2,250
**2019**	15-85+	95%	≥F0	2,000	2,000
**2020**	15-85+	95%	≥F0	1,750	1,750
**2021**	15-85+	95%	≥F0	1,500	1,125[a]
**2022**	15-85+	95%	≥F0	1,250	1,125[a]
**2023**	15-85+	95%	≥F0	1,000	1,125[a]
**2024**	15-85+	95%	≥F0	750	1,125[a]
**2025**	15-85+	95%	≥F0	500	500
**2026**	15-85+	95%	≥F0	500	500
**2027**	15-85+	95%	≥F0	500	500
**2028**	15-85+	95%	≥F0	500	500
**2029**	15-85+	95%	≥F0	500	500
**2030**	15-85+	95%	≥F0	500	500

[a] Average of 2021-2024; SVR, sustained virologic response; F, fibrosis stage

© 2020 Binka M et al. *JAMA Network Open.*

eTable 5. Sustained Virologic Response Rates Applied in the Model

This table presents the sustained virologic response rates entered into the model by time period (calendar years) for different HCV genotypes based on expert consensus and informed by published literature.

	SVR rate applied (%)[a]					
**Year**	**1990-2011**	**2012-2013**	**2014**	**2015-2016**	**2017**	**2018-2030**
**Treatment eligibility (Fibrosis stage)**	≥F2	≥F2	≥F2	≥F2	≥F2	≥F0
**G1**	40	60	85	90	95	95
**G2**	66	70	85	90	95	95
**G3**	66	70	70	90	95	95
**G4/5/6**	40	48	85	90	95	95

SVR, sustained virologic response; F, fibrosis stage; G, genotype.

[a]Based on expert consensus (CanHepC Collaborators) informed by existing literature including (but not limited to): McHutchison et al.[3], Myers et al.[4], Manns et al.[5], Ismail[6], Bachofner et al.[7], Janjua et al.[8], Darvishian et al.[9], Wilton et al.[10], as summarized by Alexopoulou et al.[11], Shafran[12], Zoratti et al.[13]

© 2020 Binka M et al. *JAMA Network Open.*

eTable 6. Treatment Scenario Assumptions Entered Into the Model

This table presents the assumptions entered for each HCV treatment scenario by waves (calendar years).

	Base	Wave 1	Wave 2	Wave 3	Wave 4	Wave 5
**Year**	**2017**	**2018**	**2019**	**2020**	**2021-2024[a]**	**2025-2030[a]**
**Age eligibility**	15-85+	15-85+	15-85+	15-85+	15-85+	15-85+
**Fibrosis eligibility**	≥F2	≥F0	≥F0	≥F0	≥F0	≥F0
**Average SVR**	95%	95%	95%	95%	95%	95%
**New diagnoses**	8,934	9,202	9,386	9,574	7,181	4,787
**New infections**	2,500	2,250	2,000	1,750	1,125	500
**Number treated**		**Optimistic**				
	12,718	10,200	10,200	10,200	10,200	10,200
		**Aggressive**				
	12,718	14,000	14,000	12,000	12,000	10,000
		**Gradual decease**				
	12,718	12,000	11,708	11,417	10,688	9,229
		**Rapid decrease**				
	12,718	12,000	11,375	10,750	9,188	6,063
		**Very aggressive**				
	12,718	14,000	14,000	14,000	14,000	14,000

[a]Average values for indicated period; SVR, sustained virologic response; F, fibrosis stage

© 2020 Binka M et al. *JAMA Network Open.*

eTable 7. Treatment scenarios: annual number of treated individuals

This table presents the annual number of treated individuals for each treatment scenario. Projected and input counts are provided, where the average projected values for 2021-2024 and for 2025-2030, respectively, were input for each of those years.

Year	Optimistic		Aggressive		Gradual decrease		Rapid decrease		Very aggressive	
	Projected	Input	Projected	Input	Projected	Input	Projected	Input	Projected	Input
**2015**	11,138	11,138	11,138	11,138	11,138	11,138	11,138	11,138	11,138	11,138
**2016**	10,496	10,496	10,496	10,496	10,496	10,496	10,496	10,496	10,496	10,496
**2017**	12,718	12,718	12,718	12,718	12,718	12,718	12,718	12,718	12,718	12,718
**2018**	10,200	10,200	14,000	14,000	12,000	12,000	12,000	12,000	14,000	14,000
**2019**	10,200	10,200	14,000	14,000	11,708	11,708	11,375	11,375	14,000	14,000
**2020**	10,200	10,200	12,000	12,000	11,417	11,417	10,750	10,750	14,000	14,000
**2021**	10,200	10,200	12,000	12,000	11,125	10,688[a]	10,125	9,188[a]	14,000	14,000
**2022**	10,200	10,200	12,000	12,000	10,833	10,688[a]	9,500	9,188[a]	14,000	14,000
**2023**	10,200	10,200	12,000	12,000	10,542	10,688[a]	8,875	9,188[a]	14,000	14,000
**2024**	10,200	10,200	12,000	12,000	10,250	10,688[a]	8,250	9,188[a]	14,000	14,000
**2025**	10,200	10,200	10,000	10,000	9,958	9,229[a]	7,625	6,063[a]	14,000	14,000
**2026**	10,200	10,200	10,000	10,000	9,667	9,229[a]	7,000	6,063[a]	14,000	14,000
**2027**	10,200	10,200	10,000	10,000	9,375	9,229[a]	6,375	6,063[a]	14,000	14,000
**2028**	10,200	10,200	10,000	10,000	9,083	9,229[a]	5,750	6,063[a]	14,000	14,000
**2029**	10,200	10,200	10,000	10,000	8,792	9,229[a]	5,125	6,063[a]	14,000	14,000
**2030**	10,200	10,200	10,000	10,000	8,500	9,229[a]	4,500	6,063[a]	14,000	14,000

[a]Average of 2021-2024 or 2025-2030, respectively.

© 2020 Binka M et al. *JAMA Network Open.*

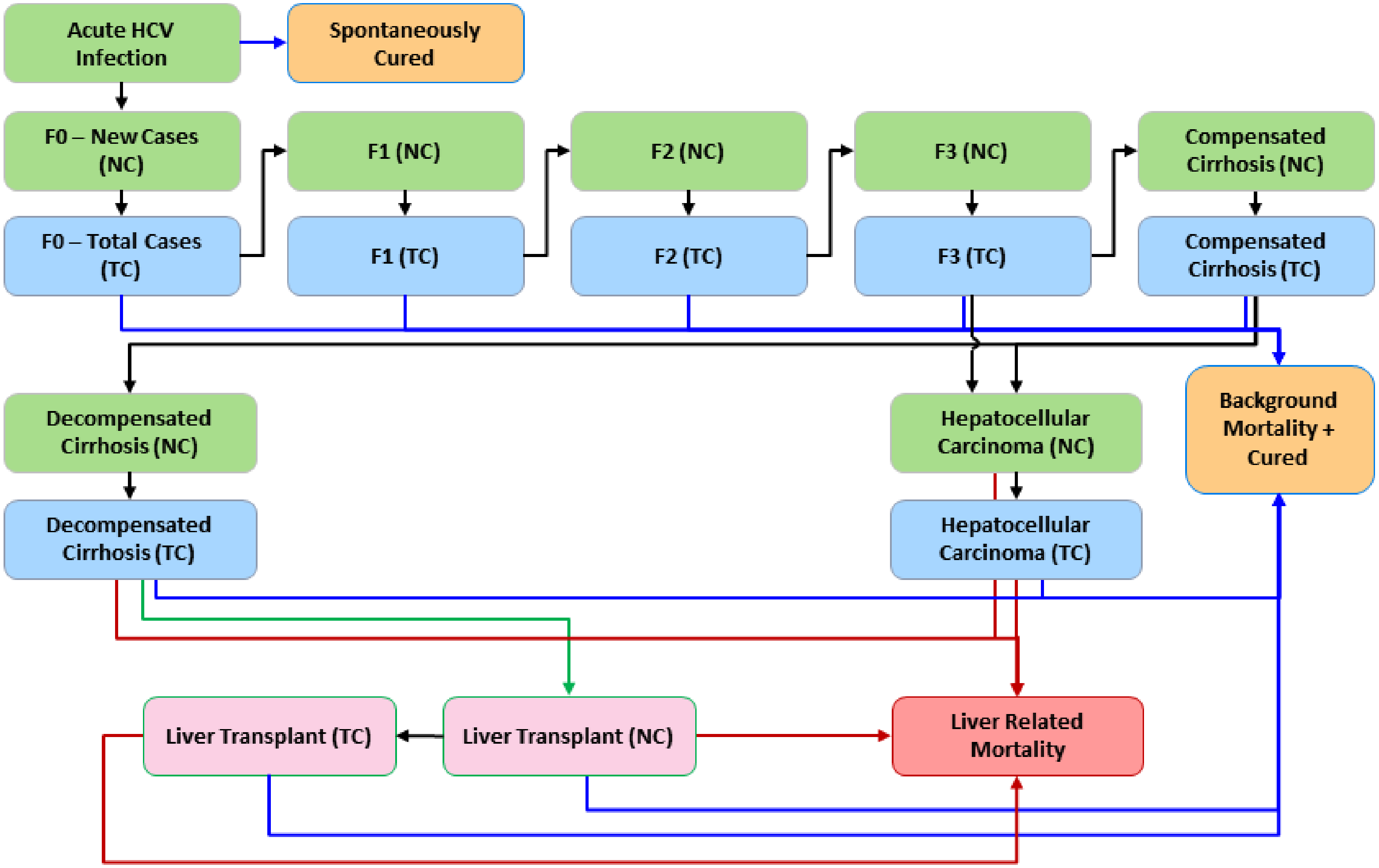

eFigure 1. Flow diagram of disease progression within the model. NC, new cases; TC, total cases. Adapted from The Lancet Gastroenterology & Hepatology, 2017, 2(3): 161-76, Blach S, Zeuzem S, Manns M, et al. Global prevalence and genotype distribution of hepatitis C virus infection in 2015: a modelling study, with permission from Elsevier and the corresponding author[14]

© 2020 Binka M et al. *JAMA Network Open.*

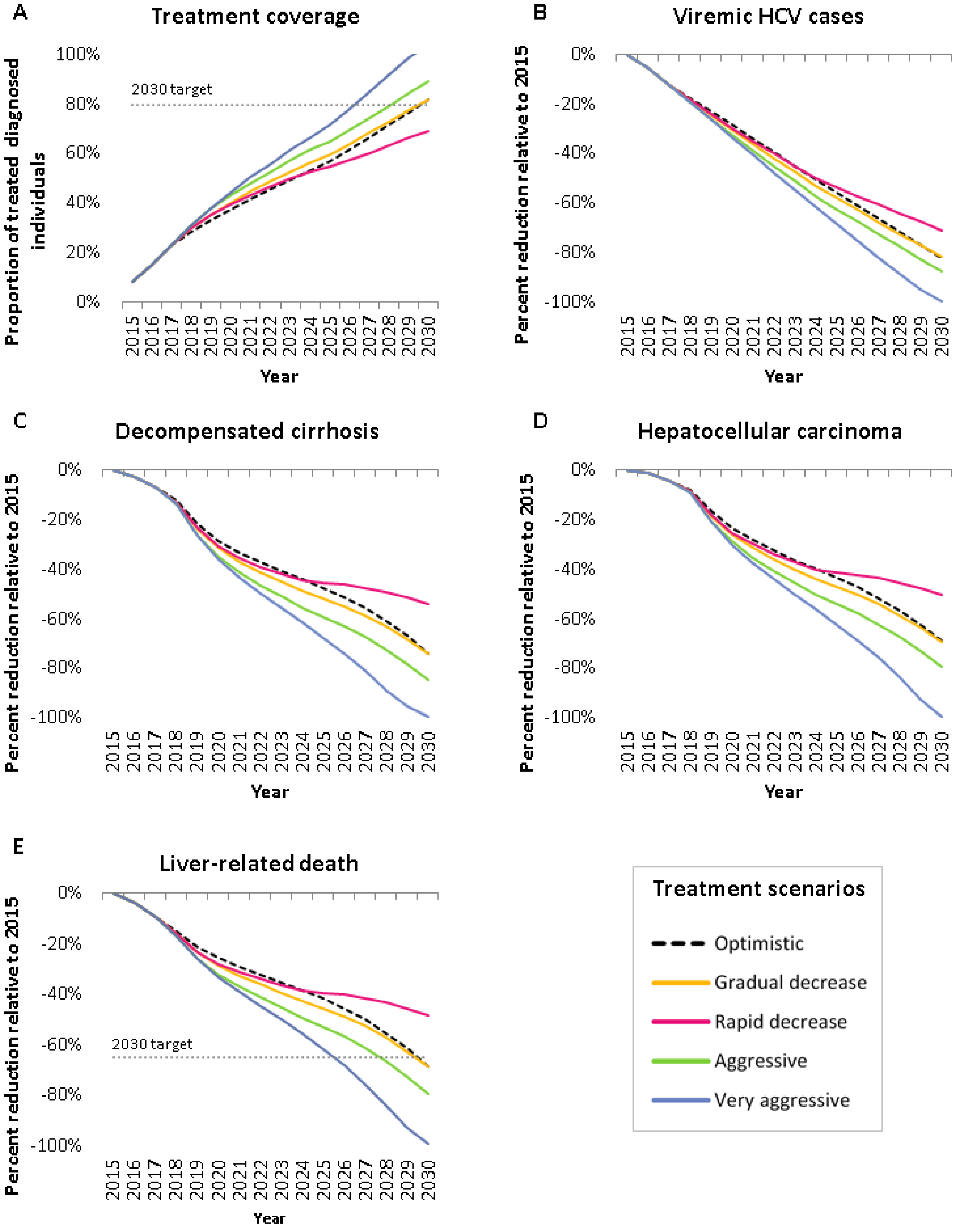

eFigure 2. Projected trends in HCV-related outcomes with each treatment scenario. Annual change in (A) HCV treatment coverage, (B) viremic HCV cases, (C) decompensated cirrhosis, (D) hepatocellular carcinoma and (E) liver-related death with different treatment scenarios. HCV: hepatitis C virus.

© 2020 Binka M et al. *JAMA Network Open.*

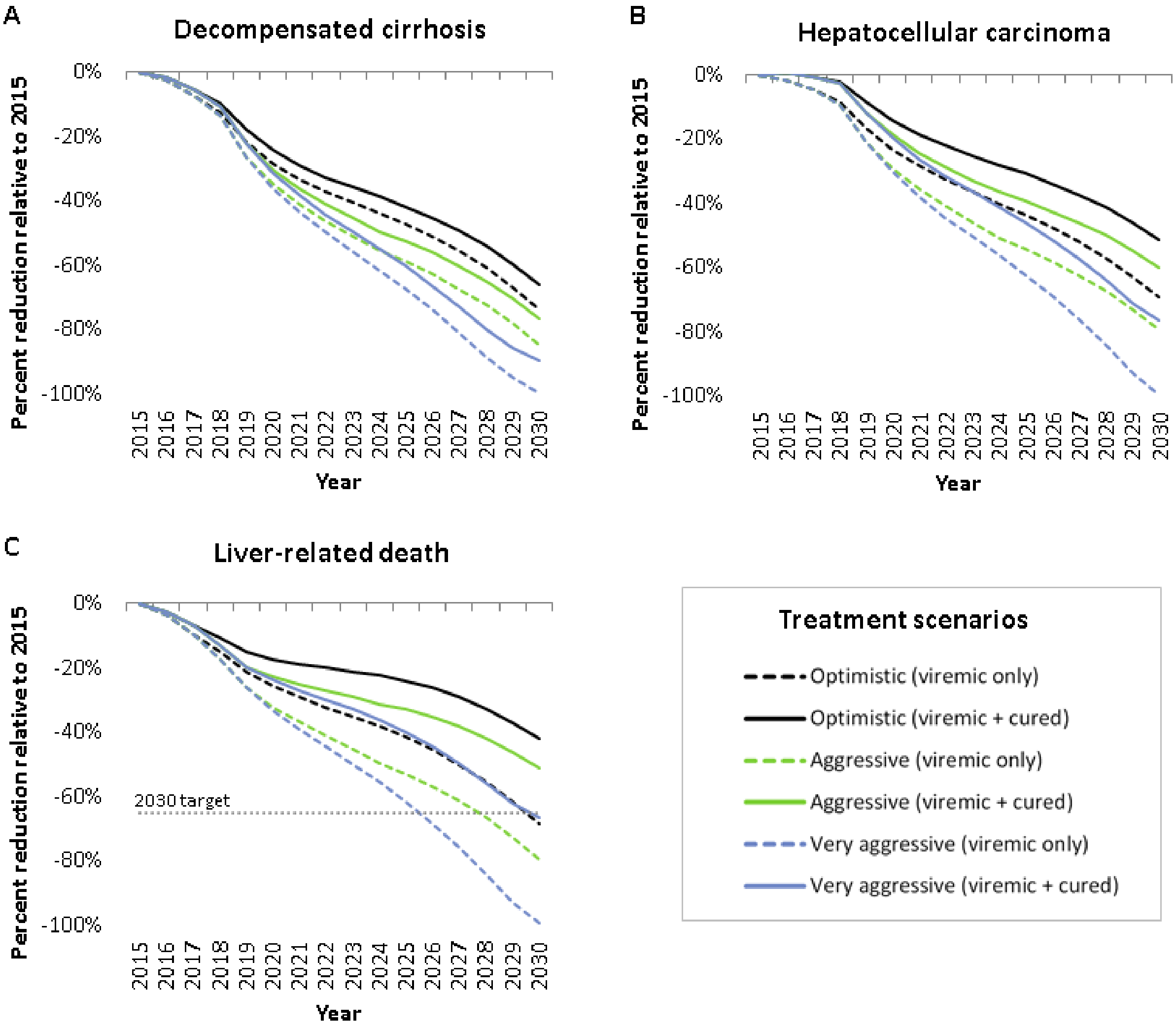

eFigure 3. The impact of advanced liver disease diagnoses among patients achieving sustained virologic response post-HCV treatment. Impact of sustained virologic response on (A) decompensated cirrhosis, (B) hepatocellular carcinoma and (C) liver-related death estimates from different treatment scenarios. Dashed lines: viremic HCV cases only; Solid lines: viremic and cured HCV cases. HCV: hepatitis C virus.

© 2020 Binka M et al. *JAMA Network Open.*

eReferences

1. United Nations Department of Economic and Social Affairs Population Division. World Population Prospects: The 2017 Revision. 2017; https://esa.un.org/unpd/wpp/Download/Standard/Population/. Accessed February 7, 2018.
2. Public Health Agency of Canada. Reported cases by age group in Canada, grouped by sex - Notifiable diseases on-line. 2017; http://diseases.canada.ca/notifiable/charts?c=abs Accessed February 7, 2018.
3. McHutchison JG, Lawitz EJ, Shiffman ML, et al. Peginterferon Alfa-2b or Alfa-2a with Ribavirin for Treatment of Hepatitis C Infection. *New England Journal of Medicine.* 2009;361(6):580-593.
4. Myers RP, Krajden M, Bilodeau M, et al. Burden of disease and cost of chronic hepatitis C virus infection in Canada. *Canadian Journal of Gastroenterology & Hepatology.* 2014;28(5):243-250.
5. Manns M, Zeuzem S, Sood A, et al. Reduced dose and duration of peginterferon alfa-2b and weight-based ribavirin in patients with genotype 2 and 3 chronic hepatitis C. *Journal of hepatology.* 2011;55(3):554-563.
6. Ismail MH. Prediction of sustained virologic responses to combination therapy of pegylated interferon-α and ribavirin in patients with chronic hepatitis C infection. *J Family Community Med.* 2013;20(1):35-40.
7. Bachofner J, Valli PV, Bergamin I, et al. Excellent outcome of direct antiviral treatment for chronic hepatitis C in Switzerland. *Swiss medical weekly.* 2018;148:w14560.
8. Janjua NZ, Darvishian M, Wong S, et al. Effectiveness of Ledipasvir/Sofosbuvir and Sofosbuvir/Velpatasvir in People Who Inject Drugs and/or Those in Opioid Agonist Therapy. *Hepatol Commun.* 2019;3(4):478-492.
9. Darvishian M, Wong S, Binka M, et al. Loss to follow-up: A significant barrier in the treatment cascade with direct-acting therapies. *Journal of Viral Hepatitis.* 2020;27(3):243-260.
10. Wilton J, Wong S, Yu A, et al. Real-world effectiveness of sofosbuvir/velpatasvir for treatment of chronic hepatitis C in British Columbia, Canada: A population-based cohort study. *Open Forum Infectious Diseases.* 2020.
11. Alexopoulou A, Karayiannis P. Interferon-based combination treatment for chronic hepatitis C in the era of direct acting antivirals. *Ann Gastroenterol.* 2015;28(1):55-65.
12. Shafran SD. HIV Coinfected Have Similar SVR Rates as HCV Monoinfected With DAAs: It's Time to End Segregation and Integrate HIV Patients Into HCV Trials. *Clinical Infectious Diseases.* 2015;61(7):1127-1134.
13. Zoratti MJ, Siddiqua A, Morassut RE, et al. Pangenotypic direct acting antivirals for the treatment of chronic hepatitis C virus infection: A systematic literature review and meta-analysis. *EClinicalMedicine.* 2020;18.
14. Blach S, Zeuzem S, Manns M, et al. Global prevalence and genotype distribution of hepatitis C virus infection in 2015: a modelling study. *The Lancet Gastroenterology & Hepatology.* 2017;2(3):161-176.

© 2020 Binka M et al. *JAMA Network Open.*
